# Coordination between water source partitioning and isohydric-anisohydric behavior shapes contrasting water use strategies of *Tamarix chinensis* and *Ulmus pumila* in saline-alkaline soils

**DOI:** 10.3389/fpls.2025.1697666

**Published:** 2025-11-26

**Authors:** Ting Wang, Chenguang Dong, Hui Wan, Rongsong Zou, Ranran Ren, Shouchao Yu, Xian Xie

**Affiliations:** 1 College of Agriculture and Biology, Liaocheng University, Liaocheng, Shandong, China; 2 Comprehensive Experimental Center in Yellow River Delta of Chinese Academy of Forestry, Dongying, Shandong, China; 3 Shandong Academy of Forestry, Jinan, Shandong, China

**Keywords:** stable isotope, water source partitioning, MixSIAR model, iso/anisohydric behavior, water-use strategy, saline-alkaline soil

## Abstract

Plant water-use strategies are key functional traits for survival in water-limited ecosystems. Understanding how water uptake coordinates with physiological characteristics in coastal saline-alkaline environments is crucial for explaining drought adaptation. However, the specific responses of these strategies to water stress in such ecosystems remain unclear. In this study, we used hydrogen and oxygen stable isotope (δ^2^H and δ^18^O) combined with a MixSIAR model to quantify seasonal variations in water uptake patterns of *Tamarix chinensis* and *Ulmus pumila* in the Yellow River Delta, China. Concurrently, we measured key physiological parameters, predawn and midday leaf water potential (Ψ_pd_, Ψ_md_) and stomatal conductance (g_s_), to assess their iso-/anisohydric behavior. Redundancy analysis (RDA) was further used to explore the coordination between water uptake patterns and iso-/anisohydric strategies. *T. chinensis* exhibited a plastic water uptake strategy, adjusting water sources with seasonal aridity, and showed anisohydric behavior characterized by larger ΔΨ and weak g_s_ sensitivity. In contrast, *U. pumila* maintained a conservative strategy, relying mainly on middle (33%) and deep soil water (31%) throughout the season and displayed isohydric behavior by tightly regulating g_s_ under declining Ψ_md_. The distinct water uptake patterns of both species were tightly coordinated with their respective iso-/anisohydric behaviors and associated physiological traits. Anisohydric plasticity in *T. chinensis* provides greater adaptability to variable precipitation, whereas the conservative isohydric strategy of *U. pumila* may reduce drought resilience. These insights can guide species selection and management to improve drought tolerance in saline-alkaline coastal plantations.

## 1 Introduction

Water availability serves as a critical determinant of vegetation growth, structure, and function in forest ecosystems (Li et al., 2022). However, forest ecosystems of coastal saline-alkaline soils face acute hydrological constraints, where seawater intrusion elevates soil and groundwater salinity, imposing severe physiological restrictions on plant development (Zhang et al., 2022; Zhu et al., 2022). Furthermore, climate-induce precipitation variability amplifies spatiotemporal heterogeneity in water availability, exacerbating the scarcity of already limited water supplies (Gessler et al., 2022; He et al., 2024). These compounding pressures inevitably intensify vegetation degradation within forest ecosystems of saline-alkaline soils (Ewe et al., 2007). The survival and growth of trees under extreme environmental variability predominantly depend on their water-use strategies and physiological characteristics (Grossiord et al., 2017; Ding et al., 2021; Bachofen et al., 2024; Morgan et al., 2025). In this context, it is a priority to better understand how plant water use strategies will be affected in the future, which improve our ecohydrological understanding of biosphere–atmosphere feedbacks and associated climate change.

The spatial and temporal dynamics in water sources absorbed by plants are generally described as water uptake patterns (Wang et al., 2017). Root water uptake is a critical drought avoidance mechanism in arid or seasonally dry habitats because it helps sustain transpiration, photosynthesis, and survival under limited precipitation and strong evaporative demand (Zhao et al., 2020; Miguez-Macho and Fan, 2021; Bachofen et al., 2024). When rainfall is scarce, deep-rooted species often shift to deeper soil layers or groundwater to meet water demand (Liu et al., 2019; Wang et al., 2022; He et al., 2024; Zhang et al., 2024). Many studies have highlighted that accessing deep water provides a competitive edge in drought-prone environments (Barbeta et al., 2015; Yang et al., 2015). In coastal saline-alkaline soils, however, salinity also exerts a strong influence on plant water uptake (Min et al., 2019; Zhang et al., 2022). Previous research shows that halophytes can draw from deeper saline soil water or groundwater, whereas non-halophytes mainly rely on low-salinity soil water or rain-derived moisture stored in the surface soil (Zhu et al., 2022). Therefore, water uptake strategies represent a crucial functional trait for enhancing plant persistence during extended droughts in plantations of saline-alkaline soils.

Variations in soil moisture regulate both the utilization of water sources and key physiological processes in trees, such as leaf water potential (Ψ) and stomatal conductance (g_s_) (Martín-Gómez et al., 2017; Ding et al., 2021; Zhao et al., 2021). The Ψ and g_s_ dynamics in sustainable forest management under global change pressures has established the isohydric/anisohydric framework as a pivotal diagnostic tool. This classification system originates from observed interspecific differences in Ψ regulation capacity under fluctuating soil water availability, whether during seasonal variations or prolonged drought (Hochberg et al., 2018). Isohydric species employ stringent stomatal optimization to minimize transpiration water loss, thereby maintaining minimal diurnal Ψ fluctuations, effectively preventing xylem cavitation-induced hydraulic failure. Conversely, anisohydric species exhibit transpiration patterns closely coupled with soil moisture dynamics, lacking defined minimum Ψ thresholds and consequently demonstrating greater ΔΨ amplitudes (Martínez-Vilalta and Garcia-Forner, 2017; Walthert et al., 2024). Moreover, anisohydric species may exhibit greater drought resistance, likely linked to access to deeper and more stable water sources (Meinzer et al., 2016; Ding et al., 2021). However, understanding the possible iso/anisohydric behaviors of halophytic and non-halophytic plants in coastal saline-alkali ecosystems have received little attention. More importantly, the coordination between water source partitioning and isohydric-anisohydric behavior as possible adaptive responses to fluctuations in soil water availability remains unclear, especially in coastal saline-alkali soils.

*Tamarix chinensis* is a perennial deciduous shrub or small tree and a primary constructive species of saline-alkali land of the Yellow River Delta. *T. chinensis* develops roots, which can enrich soil salinity and reduce soil pH (Chen et al., 2022). In addition, it contributes to soil improvement, acts as a windbreak, stabilizes sand, attenuates waves, and facilitates silt deposition. Extensive natural stands of *T. chinensis* occur in the tidal flats of the Yellow River Delta, where they play a vital role in enhancing the ecological environment and sustaining the stability of ecosystems (Sun et al., 2023). *Ulmus pumila* is a deciduous broadleaf tree species extensively utilized in ecological restoration efforts across the Yellow River Delta. In recent years, owing to the extensive exploitation of groundwater in the local area, seawater intrusion has resulted in soil salinization, and the ratio of evaporation to precipitation has escalated. Due to the shortage of freshwater resources, salt-alkali and drought stresses have emerged as the two major factors influencing the growth of vegetation in the local area. Therefore, elucidating the water use strategies of dominate species in coastal saline-alkali ecosystems constitutes a critical imperative for developing targeted mitigation measures against vegetation degradation. In this study, we used stable hydrogen and oxygen isotopes (δ^2^H and δ^18^O) to examine seasonal water uptake patterns and physiological response, in a mixed forest of *T. chinensis* and *U. pumila* in the Yellow River Delta region. We further discerned the iso/anisohydric behaviors of *T. chinensis* and *U. pumila* and coordinated them with their water uptake patterns. We addressed the following three questions: (1) Do *T. chinensis* and *U. pumila* exhibit distinct water use patterns? (2) What are the isohydric versus anisohydric behaviors of *T. chinensis* and *U. pumila*? (3) How does the coordination between water source partitioning and isohydric-anisohydric behavior shape the adaptation of *T. chinensis* and *U. pumila* to water limitations in saline-alkaline soils?

## 2 Materials and methods

### 2.1 Study area and experimental design

This study was conducted in Comprehensive Experimental Center in Yellow River Delta of Chinese Academy of Forestry (118^°^ 54′ E, 37^°^41′ N), which is situated in Dongying City, Shandong Province in east of China. The region experiences a warm-temperate monsoonal climate, characterized by pronounced seasonality. The region has a mean annual temperature of 12.4°C and receives 550 mm of precipitation annually. Rainfall is unevenly distributed, with approximately 70–80% occurring during the growing season from May to September. The soils are coastal saline-alkaline. Vegetation is dominated by xerophytic and halophytic species, along with shrubs and herbaceous plants.

*Tamarix chinensis* and *Ulmus pumila* are two dominant species in the local vegetation communities. These plantations were established around 2020 through the transplantation of two-year-old seedlings. This stand age represents a critical window of high physiological plasticity, during which water-use strategies are fundamental to species adaptation in saline-alkaline environments. The root architectures are clearly different between two woody species. In the saline-alkali soils of the Yellow River Delta, *U. pumila* exhibits a laterally extensive shallow root system, with over 80% of its fine roots concentrated within the upper 0.8m of the soil profile. In contrast, *Tamarix chinensis* develops a vertically oriented root architecture, with a mean maximum root depth of 1.2m. Three mixed forest plots (20m × 20m) were established on flat terrain in the Yellow River Delta, where *T. chinensis* and *U. pumila* are the dominant woody species. The stands are characterized by a mixed canopy with a density of approximately 5000 trees per hectare, with a species mix ratio of about 70% *T. chinensis* and 30% *U. pumila*. The understory was sparse, dominated by shallow-rooted herbaceous species such as *Phragmites australis*, *Cynanchum chinense*, *Imperata cylindrica*, *Aeluropus sinensis*.

### 2.2 Sample collection

From April to September 2024, soil, plant, rainwater, and groundwater samples were collected monthly. Rainwater was collected after each precipitation event using polyethylene bottles with funnels placed in an open area adjacent to the forest, and rainfall amounts were measured with a rain gauge. For each species, three individuals per plot were selected for xylem sampling on each sampling date. To minimize isotopic fractionation and avoid contamination by enriched water, the phloem tissue of *T. chinensis* and *U. pumila* was removed prior to analysis (Dawson and Pate, 1996). Plant samples were cut into 3–4 cm segments, sealed in screw-cap glass vials wrapped with Parafilm, and immediately frozen (−16°C) for isotope analysis. Soil samples were collected at the same time as plant tissues using a petrol-powered auger at five depths (0–20, 20–40, 40–60, 60–80, and 80–100 cm). Each soil sample was divided into two portions: one stored frozen for isotope analysis, and the other oven-dried at 80 °C to constant weight for determining gravimetric soil water content (SWC, %). Furthermore, soil electrical conductivity (EC) was determined using a 1:5 soil:water suspension and showed significant vertical stratification, with values of 1.60 ± 0.07, 1.23 ± 0.25, 1.11 ± 0.05, 0.72 ± 0.09, and 1.11 ± 0.08 dS m^-1^ for the 0–20, 20–40, 40–60, 60–80, and 80–100 cm soil layers, respectively. Groundwater was sampled from a 2m deep well located ~200 m from the plots. To prevent isotopic alteration, all samples were sealed in glass vials with Parafilm immediately after collection and stored at −16°C until analysis.

### 2.3 Stable isotopic analyses

Water from soil and plant xylem samples was extracted via cryogenic vacuum distillation. The stable isotope compositions (δ²H and δ^18^O) of rainwater, soil water, xylem water, and groundwater were determined using an isotope ratio mass spectrometer (Delta V Advantage, Thermo Fisher Scientific, Waltham, MA, USA) coupled with an elemental analyzer (Flash 2000 HT, Thermo Fisher Scientific). The analytical precision was ±1‰ for δ²H and ±0.2‰ for δ^18^O. Isotope ratios were calculated according to Equation 1:

(1) $$\text{δ }=\;[\left({\text{R}}_{\text{sample}}/\;{\text{R}}_{\text{standard}}\right)-1]\times 1000‰$$

where R_sample_ and R_standard_ denoted the ^2^H/^1^H and ^18^O/^16^O molar ratio of the sample and the V-SMOW (Vienna Standard Mean Ocean Water) standards, respectively.

### 2.4 Hydrogen isotopic offset correction

Stable water isotopes (δ^18^O and δ²H) are widely used to investigate plant water uptake strategies. Their application in plant–water relations assumes that no isotopic fractionation occurs during water absorption in terrestrial plants (Dawson and Ehleringer, 1991; Ehleringer and Dawson, 1992). However, δ²H fractionation has been observed in root water uptake for certain halophyte and xerophyte species, leading to δ²H offsets (Lin and Sternberg, 1993). To evaluate potential isotopic offsets between plant xylem water and its sources, the concepts of line-conditioned excess (LC-excess) and soil water excess (SW-excess) have been proposed by Landwehr and Coplen (2006) and further developed by Barbeta et al. (2019), respectively. In this study, groundwater, in addition to soil water, was considered an important water source, particularly in areas with shallow water tables. Therefore, a potential water line (PWL) was constructed by linear regression of soil water and groundwater isotope data (Li et al., 2021). The δ²H deviation of stem water from the PWL (PW-excess) was calculated as Equation 2:

(2) $$PW-excess\;=\;\delta^{2}H\;-a_{p}\delta^{18}O\;-b_{p}$$

where *a_p_* and *b_p_* are the slope and intercept of the PWL, respectively. Positive PW-excess values indicate that stem water δ²H is enriched relative to the PWL, with larger values reflecting greater enrichment. Conversely, negative PW-excess values indicate depletion, with more negative values representing greater depletion. A PW-excess of zero indicates no δ²H offset between stem water and the PWL. The validity of the PW-excess correction was assessed by verifying that the corrected xylem water isotopes fell within the mixing polygon defined by the potential sources (soil water and groundwater). Stem water δ²H values were corrected by subtracting the corresponding PW-excess from the original measurements and these corrected values were used in subsequent MixSIAR analysis.

### 2.5 Determination the plant water sources

The proportion of plant water uptake from each source was estimated using the Bayesian mixing model (MixSIAR), which accounts for uncertainty in root water absorption and provides a single optimal solution rather than a range of feasible outcomes (Wang et al., 2017; Zhang et al., 2024). Accordingly, MixSIAR was employed to quantify the contributions of different potential water sources to the trees.

Stem water δ²H values, corrected by the PWL (i.e., subtracting PW-excess), together with δ^18^O values, were used as the mixture data. Source data comprised the mean and standard error of soil water isotopes at various depths and groundwater. Isotope fractionation for both δ²H and δ^18^O was assumed to be zero. The Markov chain Monte Carlo (MCMC) chain length was set to ‘long’, and convergence was assessed using Gelman–Rubin and Geweke diagnostics. In the model, the error structure was specified as ‘Residual only’, and the prior was set to ‘uninformative/Generalist’ (Wang et al., 2017). Model performance was evaluated using the Akaike information criterion (AIC), Bayesian information criterion (BIC), and root mean square error (RMSE), with the dataset yielding the lowest AIC, BIC, and RMSE selected as the optimal input. The analyses were performed with *MixSIAR* package in R software (Stock and Semmens, 2013). To accurately capture the main zones of root activity and water uptake, the water sources from different soil layers were combined into shallow (0–20 cm), middle (20–60 cm) and deep (60–100 cm) soil layers to facilitate the subsequent analysis and comparison (Liu et al., 2019).

### 2.6 Leaf gas exchange and water potential measurements

Leaf water potential (Ψ) and photosynthetic performance were monitored from April to September 2024. Photosynthetic parameters were measured using a gas-exchange system (Li-6400; LiCOR Inc., Lincoln, NB, USA) with a 2cm × 3cm light-source cuvette, between 09:00 and 11:00 on cloudless days in the middle of each month. For each tree, three branches were selected, and three to five fully expanded leaves per branch were measured. Net photosynthetic rate (A_n_, μmol m^-2^ s^-1^), transpiration rate (T_r_, mmol m^-2^ s^-1^), and stomatal conductance (g_s_, mol m^-2^ s^-1^) were calculated on a leaf area basis. Predawn (Ψ_pd_, MPa) and midday (Ψ_md_, MPa) leaf water potentials were determined *in situ* using a Psypro plant water potential meter (WESCOR, USA) at 05:00–06:00 and 12:00–13:00, respectively, on sunny days in the middle of each month. Measurements were conducted on the same branches and leaves used for photosynthetic assessments.

### 2.7 Statistical analysis

Data were analyzed using two-way ANOVA analysis to examine the effects of species (*T. chinensis*, *U. pumila*), season (dry, wet), and their interaction on δ^2^H and δ^18^O values and physiological variables, followed by multiple comparisons at *p*<0.05 level of significance. Ordinary least squares (OLS) regression was employed to quantify bivariate correlations among physiological traits within each species. For multivariate analysis, redundancy analysis (RDA) was performed to evaluate the joint relationship between leaf water potential, stomatal conductance, net photosynthetic rate, transpiration rate, soil water content, and root water uptake proportion with *vegan* package in R software. All statistical analyses were performed with R 4.3.2.

## 3 Results

### 3.1 Hydrometeorological parameters

**Figure 1:**
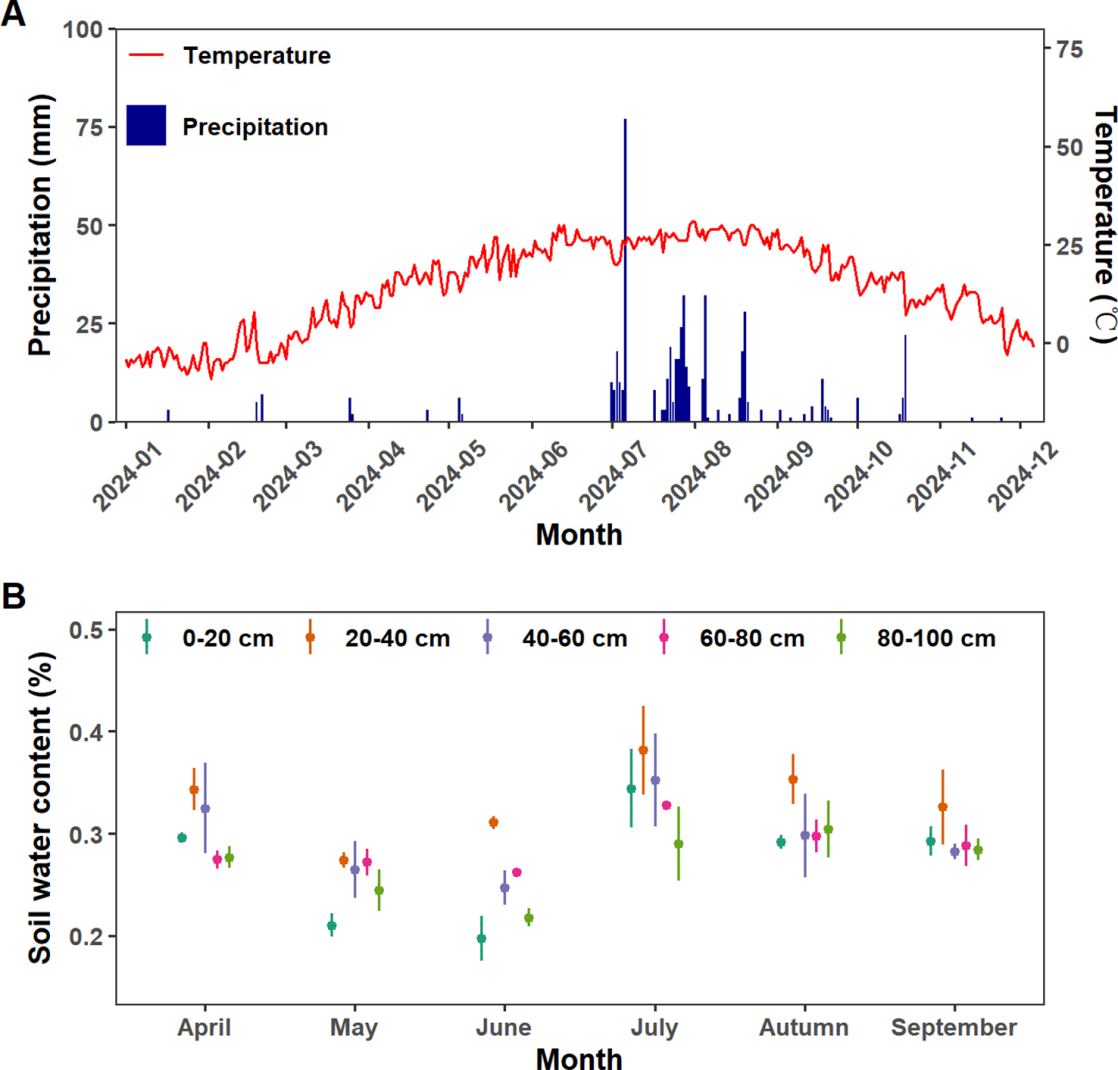
**(A)** Daily variations of precipitation and air temperature in 2024. **(B)** Monthly variations of soil water content in different layers from April to September in 2024. Different color represents soil layers.

The total precipitation was 662.5mm during the observation period in 2024 (**Figure 1A**), with pronounced seasonal variations (*p<* 0.01). The accumulated monthly precipitation during April to September was 3.0mm, 8.0mm, 0mm, 291.0mm, 270.5mm and 29.0mm, respectively (**Figure 1A**). Similarly, SWC exhibited distinct seasonal dynamics, progressively decreasing to its lowest level between April and June, sharply increasing in July, and subsequently declining (**Figure 1B**). This precipitation pattern, combined with the dynamics of SWC, defined distinct dry (April-June) and wet (July-September) seasons (**Figure 1**). The surface layer (0–20 cm) exhibited the lowest mean SWC (27.20 ± 5.57%) but highest temporal variability. Middle soil layers (20–60 cm) exhibit the highest SWC in over the entire growing season, and it was significantly influenced by seasonal variations. Although deep-layer soil moisture (60–100 cm) exhibited the highest stability, it was still significantly influenced by seasonal alternations between dry and wet periods (*p*<0.05).

### 3.2 Variations in the proportion of plant water uptake

**Figure 2:**
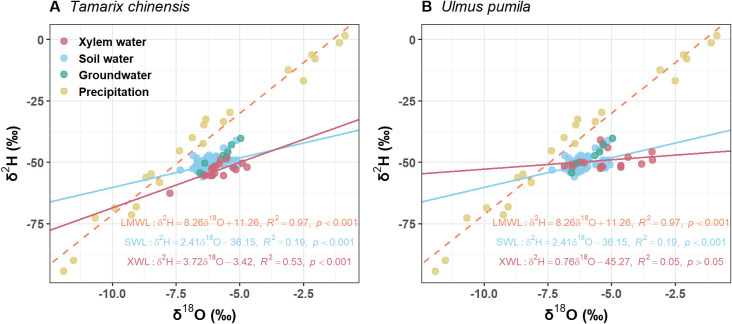
Values of δ^2^H and δ^18^O from **(A)***Tamarix chinensis* and **(B)***Ulmus pumila* from April to September. LMWL is the local meteoric line (δ^2^H =8.26 δ^18^O +11.26, R^2^=0.97, *p*<0.01) based on the isotopic values of the precipitation. SWL is soil water line which is fitted based on the isotopic values of soil water. XWL is xylem water line which is fitted based on the isotopic values of xylem water.

Precipitation δ²H and δ^18^O values were distributed along a line with a slope of 8.25 and an intercept of 11.26, representing the local meteoric water line (LMWL) (**Figure 2**). Most soil water samples plotted to the lower right of the LMWL, forming a soil water line (SWL) with a slope of 2.41 (**Figure 2**). Across the growing season, surface soil water was significantly more enriched than deeper layers (*p*<0.05 for both isotopes), reflecting evaporative effects at the soil surface (**Figure 2**). The isotopic signatures of *U. pumila* closely aligned with those of soil water, suggesting that it primarily utilized water from different soil depths (**Figure 2B**). By contrast, the isotopic values of *T. chinensis* fell outside the soil mixing space, indicating potential hydrogen isotope fractionation during water uptake (**Figure 2A**).

**Figure 3:**
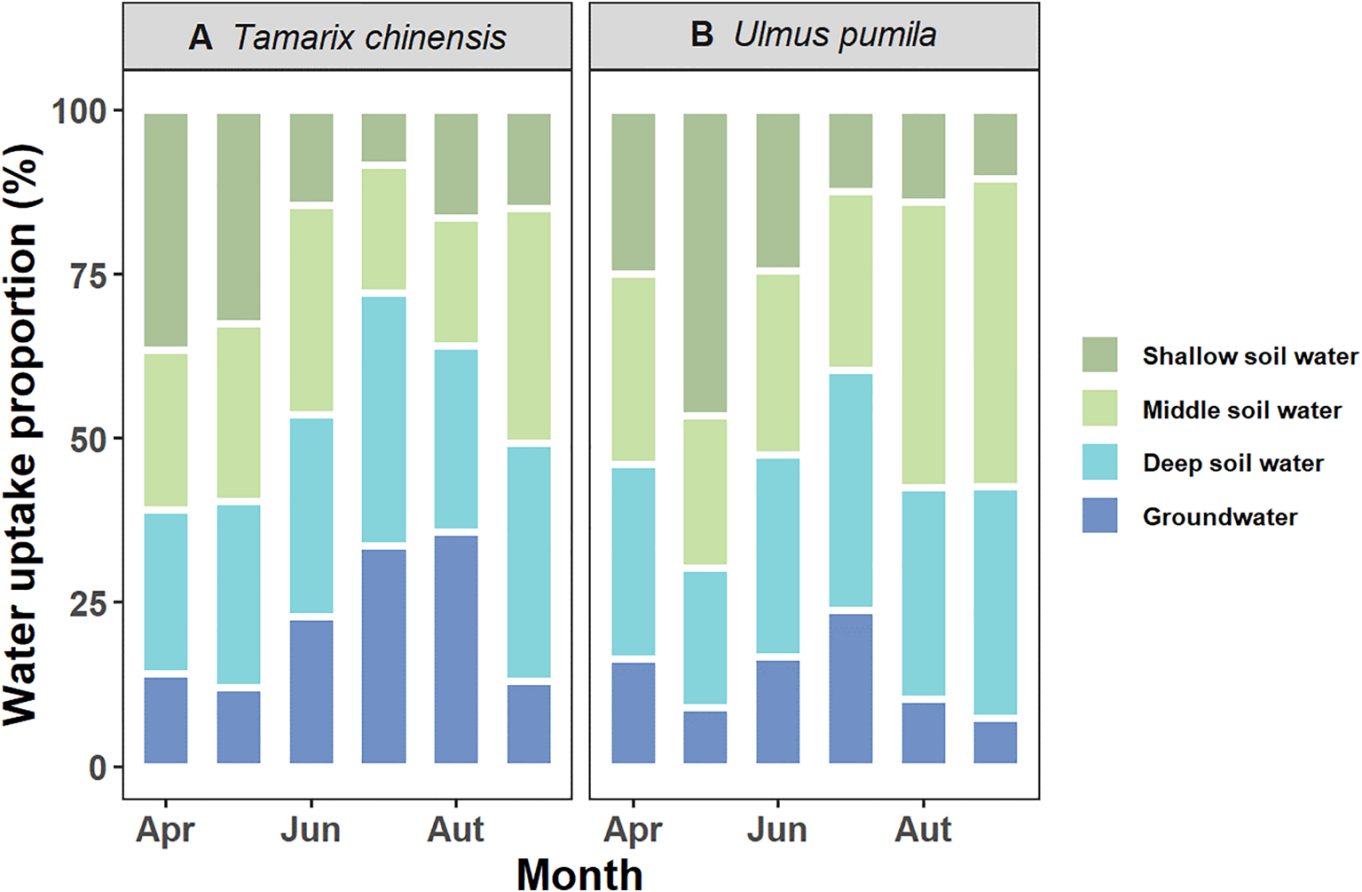
Seasonal variations in water uptake proportions for **(A)***Tamarix chinensis* and **(B)***Ulmus pumila* using MixSIAR from April to September.

Dual-isotope (δ^2^H and δ^18^O) with MixSIAR modeling revealed contrasting water uptake patterns between *T. chinensis* and *U. pumila* (**Figure 3**). *T. chinensis* exhibited marked seasonal shifts in water source partitioning. It primarily relied on shallow and intermediate soil water during the early growing season (61% and 60% in April and May, respectively), but progressively increased deep soil water and groundwater utilization to 54% in June and 72% in July as drought intensity escalated (**Figure 3A**). In wet season (August-September), *T. chinensis* tend to uptake water from intermediate and deep soil water, with the contributions rebounding to 48% and 72%, respectively (**Figure 3A**). In contrast, *U. pumila* maintained stable reliance on intermediate (33%) and deep (31%) soil layer water throughout the growing season, with limited groundwater exploitation (14%, **Figure 3B**). Collectively, *T. chinensis* employs a plastic water uptake strategy, dynamically shifting soil water acquisition depths in response to seasonal drought, whereas *U. pumila* maintains a conservative water-use pattern characterized by stable dependence on middle and deep soil water throughout the growing season.

### 3.3 Estimates of isohydric and anisohydric behavior

**Figure 4:**
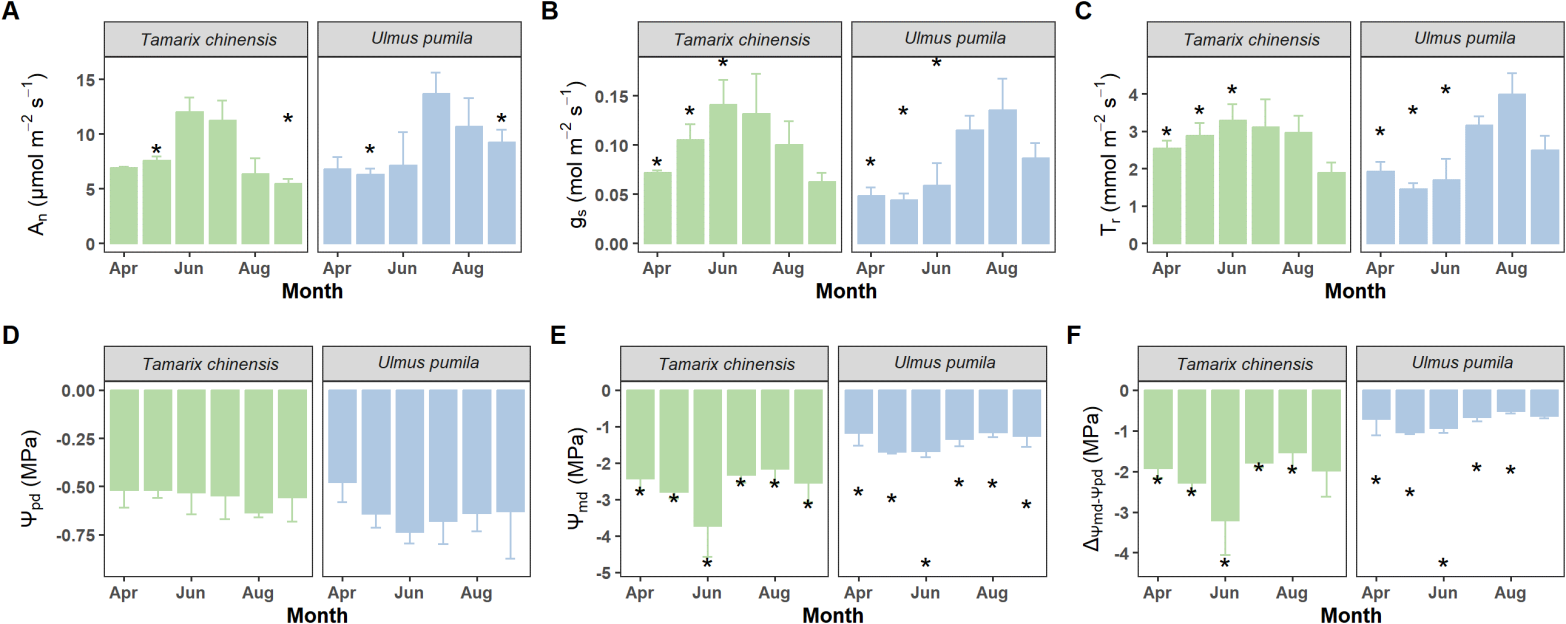
Monthly variations of net photosynthetic rate (A_n_, **A**), stomatal conductance (g_s_, **B**), transpiration rate (T_r_, **C**), predawn leaf water potential (Ψ_pd_, **D**), midday leaf water potential (Ψ_md_, **E**) and the difference between midday and predawn leaf water potential (Δ_Ψmd-Ψpd_, **F**) for *Tamarix chinensis* and *Ulmus pumila* from April to September 2024. The asterisks on the columns indicate that there are significant differences in the physiological characteristics of *Tamarix chinensis* and *Ulmus pumila* at *p*<0.05. Error bars express as standard deviation.

*T. chinensis* and *U. pumila* exhibited contrasting water use strategies (**Figure 4**; **Table 1**). *T. chinensis* exhibited higher A_n_ than *U. pumila* in dry season (May), but lower A_n_ in wet season (September; **Figure 4**). g_s_ in *T. chinensis* was significantly 32% higher than *U. pumila* during dry season (April−June), with no divergence in the wet season (July-September; **Figure 4B**). Similarly, T_r_ was significantly 41% greater in *T. chinensis* than in *U. pumila* under drought stress (**Figure 4C**). Although both species showed comparable Ψ_pd_ (**Figure 4D**), *T. chinensis* maintained significantly lower Ψ_md_ than *U. pumila* across growing season (**Figure 4E**). Similarly, the ΔΨ was significantly smaller in *T. chinensis* than in *U. pumila* during growing season (**Figure 4F**), highlighting their fundamentally divergent water-use strategies.

**Table 1:** Xylem water isotopes and physiological traits of *Tamarix chinensis* and *Ulmus pumila* across dry (April-June) and wet (July-September) seasons.

Parameter	Dry season		Wet season	
	*Tamarix chinensis*	*Ulmus pumila*	*Tamarix chinensis*	*Ulmus pumila*
δ²H (‰)	-54.63 ± 1.17 Ab	-50.43 ± 0.75 Aa	-51.55 ± 0.91 Aa	-48.17 ± 1.43 Aa
δ^18^O (‰)	-5.72 ± 0.30 Ab	-4.49 ± 0.27 Aa	-5.94 ± 0.10 Aa	-6.06 ± 0.20 Ba
A_n_ (μmol m^-2^ s^-1^)	8.83 ± 0.83 Aa	6.75 ± 0.56 Bb	7.70 ± 0.98 Ab	11.21 ± 0.88 Aa
T_r_ (mmol m^-2^ s^-1^)	2.90 ± 0.15 Aa	1.69 ± 0.13 Bb	2.65 ± 0.25 Aa	3.21 ± 0.25 Aa
g_s_ (mol m^-2^ s^-1^)	0.10 ± 0.01 Aa	0.05± 0.01 Bb	0.10 ± 0.01 Aa	0.11 ± 0.01 Aa
Ψ_pd_ (MPa)	-0.52 ± 0.03 Aa	-0.62 ± 0.04 Aa	-0.58 ± 0.03 Aa	-0.65 ± 0.05 Aa
Ψ_md_ (MPa)	-2.98 ± 0.25 Bb	-1.51 ± 0.10 Aa	-2.34 ± 0.13 Ab	-1.25 ± 0.07 Aa

Different uppercase letters indicate significant differences between seasons (*p*<0.05), and different lowercase letters indicate significant differences between species within the same season (*p*<0.05).

Values are presented as mean ± SE.

**Figure 5:**
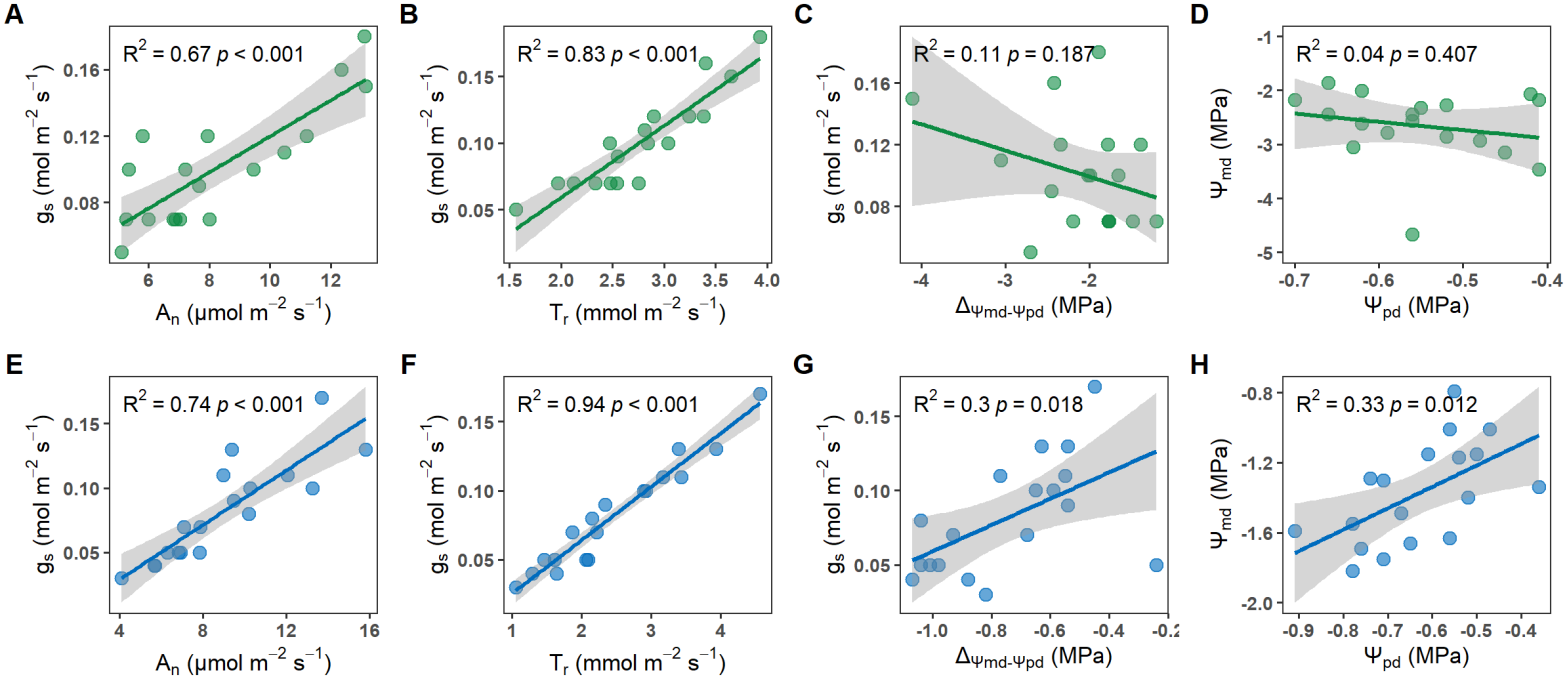
Relationship between stomatal conductance (g_s_) and net photosynthetic rate (A_n_; **A, E**), stomatal conductance (g_s_) and transpiration rate (T_r_; **B, F**), stomatal conductance (g_s_) and the difference between predawn and midday leaf water potential (ΔΨ; **C, G**), predawn (Ψ_pd_) and midday leaf water potential (Ψ_md_; **D, H**) for *Tamarix chinensis* and *Ulmus pumila* from April to September in 2024.

Crucially, *T. chinensis* and *U. pumila* exhibited contrasting isohydric and anisohydric behaviors, evident in their water potential regulation (**Figure 5**). For *U. pumila*, Ψ_pd_ positively correlated strongly with Ψ_md_ (R² = 0.68, *p*<0.05; **Figure 5E**), and ΔΨ increased significantly with g_s_ (R² = 0.30, *p*<0.05; **Figure 5F**), collectively demonstrating tight stomatal regulation of leaf water status characteristic of isohydric behavior. In contrast, *T. chinensis* showed no correlation between Ψ_pd_ and Ψ_md_ (**Figure 5C**) or between g_s_ and ΔΨ (**Figure 5D**). These distinct hydraulic responses confirm that *T. chinensis* demonstrates strong anisohydric behavior, while *U. pumila* exhibits characteristic isohydric regulation.

### 3.4 Water uptake depth coordinated with iso/anisohydric behavior

**Figure 6:**
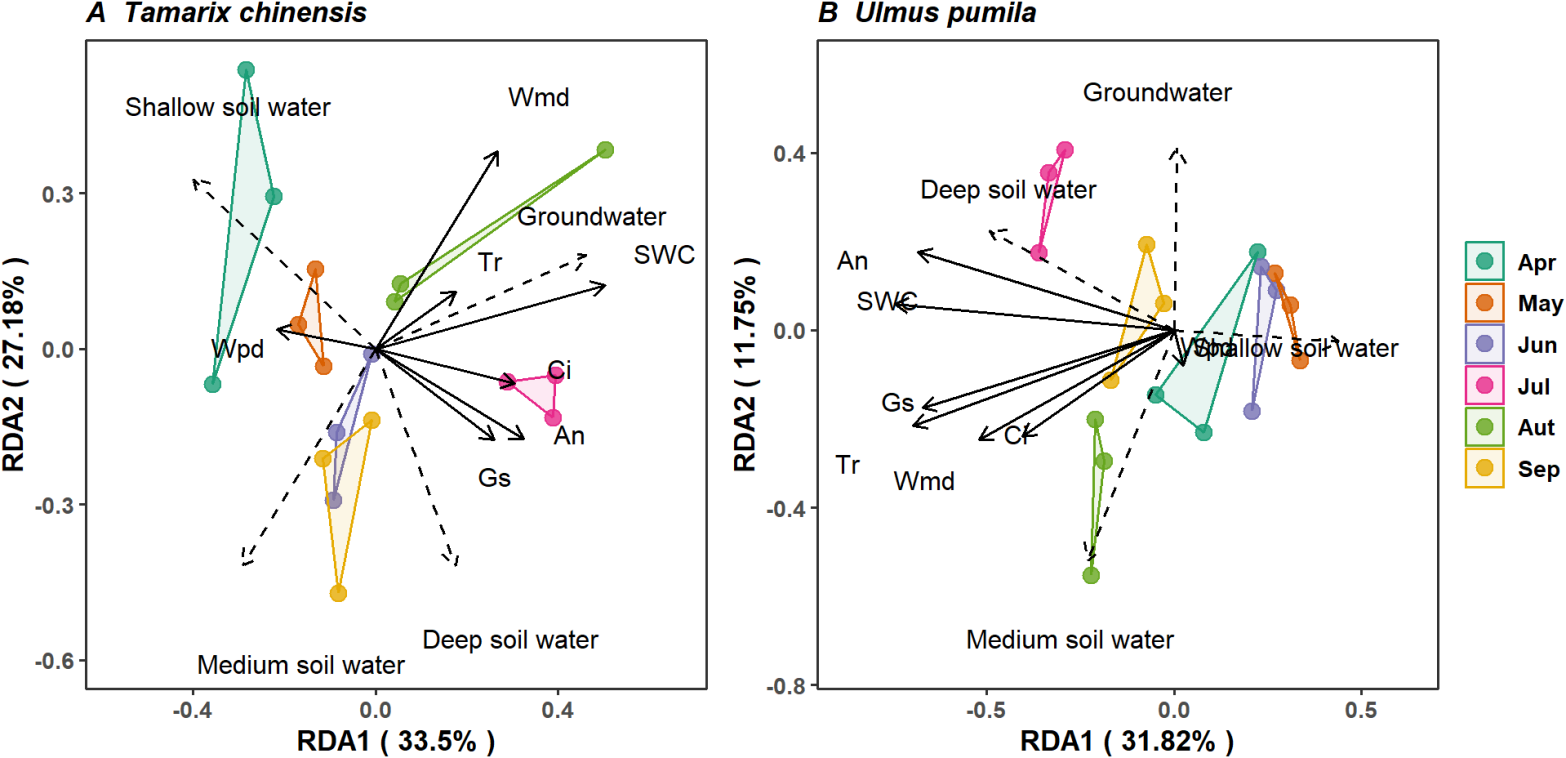
Correlation between water uptake from each source and basic topography, soil properties for *Tamarix chinensis***(A)** and *Ulmus pumila***(B)** in redundancy analysis. Solid line denotes the explained variables. Ψ_pd_ is predawn leaf water potential; Ψ_md_ midday leaf water potential; ΔΨ the difference between predawn and midday leaf water potential; g_s_ stomatal conductance; and A_n_ net photosynthetic rate, T_r_ transpiration rate and SWC soil water content.

RDA revealed that the main components explained 60.68% and 43.57% of the total variance in water source utilization for *T. chinensis* and *U. pumila*, respectively (**Figure 6**), highlighting the coordination of physiological characters and water uptake patterns. For *T. chinensis*, g_s_, Ψ_md_, and C_i_ collectively dominated water uptake variations, with reductions in Ψ_md_ and g_s_ correlating with increased dependence on deep soil water (**Figure 6A**). In contrast, *U. pumila* exhibited stronger coupling between A_n_, T_r_, g_s_ and SWC, where maintained middle and deep soil water utilization corresponded to high A_n_ and T_r_ and SWC availability (**Figure 6B**). This indicates *U. pumila* maintained water source utilization despite constrained photosynthetic assimilation. Collectively, water source partitioning is coordinate with isohydric-anisohydric behavior of *T. chinensis* and *U. pumila* in saline-alkaline soils.

## 4 Discussion

### 4.1 Differences in water uptake patterns between *T. chinensis* and *U. pumila*

Understanding the water uptake patterns influencing drought vulnerability is crucial for effective forest management and vegetation restoration strategies (Miguez-Macho and Fan, 2021; Bachofen et al., 2024; He et al., 2024; Wang et al., 2024). Isotope mixing models revealed contrasting water uptake pattern between *T. chinensis* and *U. pumila* (**Figure 3**). *U. pumila* exhibited a conservative strategy with minimal seasonal variation, predominantly relying on stable middle and deep soil water throughout the growing season (**Figure 3**). This may attribution that soil salinity plays a key role in shaping plant water uptake strategy in saline-alkaline soils (Zhang et al., 2022; Zhu et al., 2022). Surface soil water is prone to rapid evaporation, leading to salt accumulation in upper layers (Xia et al., 2019; Lei et al., 2025). Meanwhile, groundwater in the study area exhibited high salinity levels, which can inhibit root penetration due to osmotic stress (Sun et al., 2022). As a result, *U. pumila* roots tend to avoid both the saline surface layer and the high-salinity groundwater, further reinforcing their dependence on middle and deep soil water. This avoidance strategy reflects a form of vertical confinement, where the plant minimizes upward or downward root extension to evade salt stress, while maximizing horizontal spread within the favorable mid-soil zone (Sun et al., 2023). This “horizontal expansion-vertical limitation” pattern restricts the tree’s ability to tap into deeper water reserves during prolonged droughts, potentially increasing its vulnerability under extreme water stress.

In contrast, *T. chinensis* demonstrated pronounced seasonal plasticity in water source partitioning. *T. chinensis* progressively increased reliance on deep soil water and groundwater relatively dry conditions, but shifting to middle and deep soil water under wet conditions (e.g. August and September) (**Figure 3**). This pattern of water uptake aligns with earlier studies reporting that halophytes adjust their water sources between shallow and deep soils in response to changes in soil water availability (Sun et al., 2022; Zhu et al., 2022). Shifting in water uptake between shallow and deep soil layers are primarily determined by a species’ root dimorphism (Yang et al., 2015; Wang et al., 2017; Liu et al., 2019). *T. chinensis* is one such species, with a root architecture composed of both shallow and deep roots, allowing it to extract water from multiple soil layers depending on environmental conditions (Imada et al., 2015; Sun et al., 2022). Besides, *T. chinensis* uptake more high-salinity groundwater than *U. pumila*, demonstrating exceptional salt tolerance that facilitates root extension into saline aquifers. This ability to dynamically adjust water use in response to fluctuations in rainfall and soil moisture, characterized by a shift in uptake from shallow to deeper soil layers, reflects strong ecological plasticity, which is crucial for plant survival in variable and often harsh environments and is often associated with a greater capacity to adapt to changing conditions (Grossiord et al., 2017; Jiang et al., 2020; Gessler et al., 2022; Wang et al., 2022). Therefore, the differences in root distribution and salt tolerance underpin *T. chinensis*’s superior drought resistance.

### 4.2 Contrasting isohydric and anisohydric behavior between *U. pumila* and *T. chinensis*

Our findings demonstrate contrasting iso-/anisohydric behaviors between *U. pumila* and *T. chinensis*, which reflect their fundamentally different strategies for regulating plant water use under fluctuating environmental conditions. *U. pumila* exhibited a typical isohydric response, characterized by tight control of stomatal conductance. Specifically, we observed significant positive correlations between *U. pumila* g_s_ and Ψ_md_ (R² = 0.33, *p*=0.012; **Figure 5H**), as well as between g_s_ and the diurnal water potential difference (ΔΨ, Ψ_md_ – Ψ_pd_) (R²= 0.30, *p*=0.018; **Figure 5G**). This indicates that *U. pumila* conserves water during drought by closing stomata in response to declining leaf water potential, reflecting a classic isohydric strategy of transpiration control. Similar results suggested that some plant species employed an isohydric strategy based on daily changes in stomatal conductance and stem vulnerability (McDowell et al., 2008; Meinzer et al., 2016; Martín-Gómez et al., 2017). This reinforces the general understanding that isohydric plants adjust stomatal behavior to reduce water loss, with stomatal conductance responding directly to changes in water potential (Martínez-Vilalta and Garcia-Forner, 2017; Hochberg et al., 2018). In contrast, *T. chinensis* demonstrated an anisohydric behavior, as evidenced by the lack of significant correlation between g_s_ and ΔΨ or Ψ_md_ (**Figure 5C** and D) and more negative ΔΨ (Ψ_md_ – Ψ_pd_) (**Figure 4F**). During dry months (April–June), *T. chinensis* maintained higher g_s_ and T_r_ than *U. pumila* (**Figures 4B, C**), despite exhibiting significantly lower Ψ_md_ (**Figure 4E**). This suggests that *T. chinensis* allows greater fluctuation in water potential while sustaining gas exchange, even under increasing drought intensity. Such behavior is adaptive in environments with temporally variable water availability, as it enables continuous carbon assimilation at the cost of increased risk of hydraulic failure (Hochberg et al., 2018; Xue et al., 2024).

Furthermore, seasonal variance in Ψ_md_ provided additional evidence of these divergent strategies. *T. chinensis* exhibited larger Ψ_md_ values under drought, while *U. pumila* maintained smaller Ψ_md_ across seasons (**Figure 4F**). The sharp rebound in Ψ_md_ for *T. chinensis* in July, coinciding with the onset of the wet season, further indicates its capacity for rapid hydraulic recovery, consistent with anisohydric flexibility. This result aligns with Franks et al. (2007), who observed that Ψ_md_ in anisohydric plants exhibits seasonal fluctuations, highlighting the need to evaluate iso- and anisohydric behaviors across multiple temporal scales, as performed in the present study. Taken together, these results suggest that *U. pumila* adopts a conservative, water-saving approach under stress, prioritizing hydraulic safety, whereas *T. chinensis* tolerates more negative water potentials to maintain higher gas exchange, reflecting a riskier but potentially more productive strategy. This divergence in iso-/anisohydric behavior underpins each species’ unique drought adaptation strategy in the saline-alkaline ecosystems of the Yellow River Delta.

### 4.3 Coordination between water uptake patterns and iso-/anisohydric behavior

**Figure 7:**
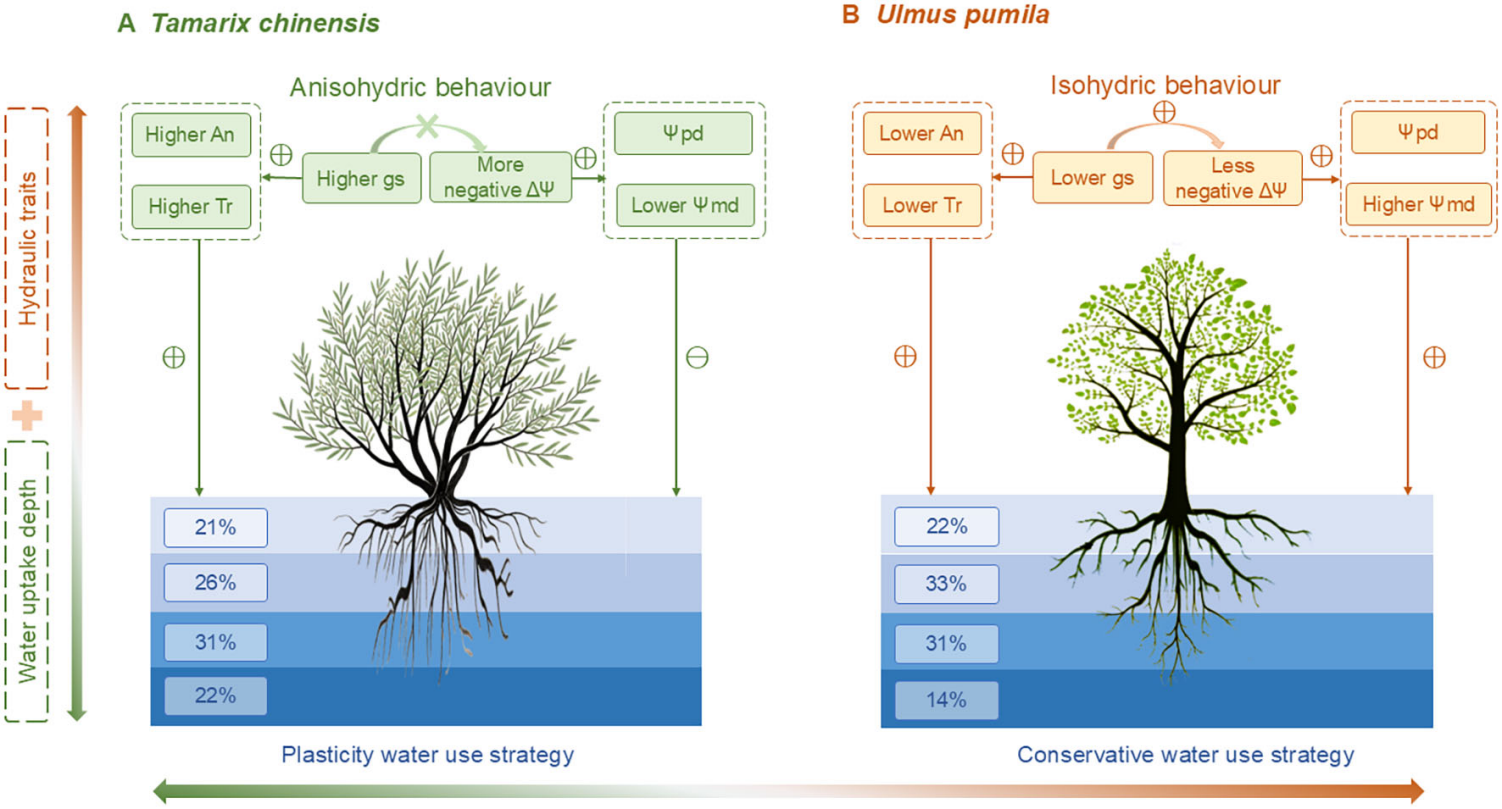
Conceptual framework illustrating contrasting water use strategies of *Tamarix chinensis***(A)** and *Ulmus pumila***(B)** under drought. *T. chinensis* exhibits an anisohydric behavior characterized by higher photosynthetic rate (A_n_), transpiration (T_r_), stomatal conductance (g_s_), resulting in both a more negative water potential difference (ΔΨ) and lower midday water potential (Ψ_md_), which lead to greater plasticity in water uptake depth. In contrast, *U. pumila* shows an isohydric behavior with lower A_n_, T_r_, g_s_, and a less negative ΔΨ, and higher Ψ_md_, adopting a conservative water use strategy with relatively stable, middle soil water uptake. Hydraulic traits (upper panel) and water uptake depth proportions (lower panel) jointly explain *Tamarix chinensis* and *Ulmus pumila* divergent water use strategies.

The water uptake patterns of *T. chinensis* and *U. pumila* are tightly coordinated with their respective iso-/anisohydric behaviors and associated physiological traits (**Figures 6**, **7**). Redundancy analysis (RDA) revealed that water uptake depth and physiological regulation co-vary in response to environmental conditions, suggesting a functional integration of water-use strategy and hydraulic traits (**Figure 6**). For *U. pumila*, the increased reliance on deep soil water was associated with higher Ψ_md_, g_s_, A_n_, T_r_ and SWC (**Figure 6B**). These physiological traits, in coordination with isohydric regulation, constrain water source selection by limiting water uptake to relatively stable, less saline soil layers in order to maintain leaf water potential under drought conditions (Ding et al., 2021; Zhu et al., 2022). Although *U. pumila* demonstrates capacity to extract water from middle and deep soil layers, its limited salt tolerance and shallow root penetration inhibit its ability to exploit high-salinity groundwater in saline-alkaline environments. This physiological constraint likely restricts its drought resilience under prolonged or severe aridity.

In contrast, *T. chinensis* exhibited increased reliance on deep soil water during the dry season was associated with lower Ψ_md_ and higher g_s_ (**Figure 6A**). This response supports that anisohydric strategy, characterized by sustained stomatal opening and declining water potential, facilitates access to more stable, deeper water reserves (Kannenberg et al., 2019; Walthert et al., 2024). This physiological coordination supports the view that anisohydric regulation enhances the ecological plasticity of *T. chinensis*, allowing it to adjust water uptake in response to temporal changes in soil moisture. Indeed, our findings show that *T. chinensis* dynamically shifted its water source from shallow to deep layers as drought intensified (**Figure 3A**). The physiological traits support a flexible drought response strategy, maximizing water acquisition and gas exchange even under prolonged water deficits.

Therefore, our findings suggest that the coordination between iso-/anisohydric behavior and root water uptake strategy enables *T. chinensis* and *U. pumila* to adopt contrasting yet functional drought responses. *T. chinensis* exemplifies an anisohydric species with high physiological plasticity, able to flexibly shift water sources while maintaining gas exchange under stress. In contrast, *U. pumila* exemplifies a conservative isohydric plant, whose exploit middle and deep soil water sources may render it more vulnerable under future drought intensification and soil salinization aggrandizement. This coordination between water-use pattern and physiological traits provides a valuable framework for understanding species-specific drought resilience in water-limited ecosystems.

### 4.4 Implication for forest management in saline-alkaline ecosystem

The Yellow River Delta is a unique coastal ecosystem where vegetation growth is constrained by shallow groundwater tables, high soil salinity, and strong seasonal variability in precipitation (Liu et al., 2018; Xia et al., 2019; Zhang et al., 2022). Prolonged droughts exacerbate salt accumulation in the upper soil layers through enhanced evaporation, while freshwater scarcity limits leaching, creating a dual stress of salinity and water deficit for plant survival (Hassani et al., 2021; Chen et al., 2022). In this context, understanding the contrasting water-use strategies of *T. chinensis* and *U. pumila* offers valuable guidance for afforestation and restoration planning in saline-alkaline environments.

Our study shows that *U. pumila* adopts an isohydric strategy, maintaining stable leaf water potentials through tight stomatal control and a consistent dependence on intermediate and deep soil water. This conservative water use strategy reduces hydraulic risk but tends to be more vulnerable to water stress especially in the highly seasonal precipitation regimes of the Yellow River Delta. First, the isohydric behavior of *U. pumila* indicates reliance on carbohydrate reserves to satisfy carbon requirements for respiration and osmoregulation. Continuous metabolic demand may progressively deplete these reserves, ultimately resulting in carbon starvation (McDowell et al., 2008; Skelton et al., 2015; Kono et al., 2019). Such depletion progressively undermines drought resilience, reducing stand productivity and ecosystem health (Adams et al., 2017; Hartmann et al., 2018). Besides, overreliance on a single water source can intensify hydrological trade-offs, amplifying water acquisition challenges when that source is depleted (Yin et al., 2024). Consequently, Although *U. pumila*’s isohydric behavior may provide an adaptive strategy to survive in water-limited environment, the long-term viability of this strategy is questionable. Balancing ecological benefits with hydrological sustainability requires optimizing planting densities and implementing rigorous monitoring of deep soil water reserves.

*T. chinensis* demonstrates high ecological plasticity through its anisohydric behavior and flexible shifts in water uptake between shallow, deep, and groundwater in response to water variability. This adaptability sustains higher gas exchange during drought and allows exploitation of deep soil water reserves when surface soils desiccate or salinity rises. These traits that position *T. chinensis* as a key species for revegetating degraded saline-alkaline zones, particularly in drought-prone regions. Notably, *T. chinensis* outcompetes *U. pumila* in such environments by flexible shifting in water uptake, supporting superior productivity during seasonal water fluctuations. Although anisohydric plants may face a higher risk of hydraulic failure, their flexibility in water sourcing enables them to sustain relatively greater water acquisition (Martínez-Vilalta and Garcia-Forner, 2017; Walthert et al., 2024). This adaptable water-use strategy is likely to confer greater advantages under the anticipated increase in drought frequency and will promote tree’s survival probability (Muñoz-Gálvez et al., 2024).

Our study reveals a tight coordination between dynamic water source partitioning and distinct iso-/anisohydric behaviors in two dominant tree species under saline-alkaline stress. This integrated approach addresses a critical gap by uncovering the mechanistic link between root water uptake and stomatal regulation in saline-alkaline soils, thereby providing a more holistic understanding of their drought adaptation strategies. Our results suggest that afforestation and restoration in the Yellow River Delta should account for the interaction between species-specific water-use strategies, soil salinity dynamics, and water availability. *T. chinensis* appears better suited than *U. pumila* for initiating revegetation in the Yellow River Delta. *T. chinensis*, with its anisohydric behavior and flexible water uptake strategy, should be prioritized for afforestation in areas with high seasonal variability in precipitation and soil salinity. Its ability to utilize deeper, more saline water sources confers greater resilience to drought. *U. pumila* may be better suited for planting in areas with more reliable freshwater availability or in mixed-species plantations. Its isohydric conservatism could be complemented by the plasticity of species like *T. chinensis*, potentially enhancing overall ecosystem stability and resource partitioning. Nevertheless, determining an optimal planting density may be necessary to avoid excessive depletion of deep soil water resources. Aligning forest management with species-specific water-use strategies, such as the anisohydric plasticity of *T. chinensis* versus the isohydric conservatism of *U. pumila*, is crucial for enhancing drought resilience in seasonally arid forests.

## 5 Conclusion

Differences in water uptake patterns and physiological responses were observed between *T. chinensis* and. *U. pumila*. *T. chinensis* employs a plastic water uptake strategy, dynamically shifting soil water acquisition depths in response to seasonal aridity, whereas *U. pumila* maintains a conservative water-use strategy characterized by stable dependence on predictable moisture reservoirs throughout the growing season. *T. chinensis* is an anisohydric species, as characterized by a greater diurnal water potential range and a lack of significant correlation between gs and water potential (*p>*0.05). In contrast, the isohydric *U. pumila* had a more conservative strategy, tightly regulating transpiration by closing stomata in response to declining leaf water potential (*p<*0.05). The water uptake patterns of *T. chinensis* and *U. pumila* are tightly coordinated with their respective iso-/anisohydric behaviors and associated physiological traits. *T. chinensis* facilitates access to more stable, deeper water reserves to sustain stomatal opening and declined water potential. The physiological characteristics of *U. pumila*, in conjunction with isohydric regulation, maintain leaf water potential under drought conditions by restricting water uptake exclusively to the relatively stable and low-salinity middle and deep soil layers. Our findings demonstrate that, for saline-alkali soils, *T. chinensis*’s water-use strategy conveys stronger adaptive advantages under shifting precipitation regimes. Aligning forest management with species-specific water-use strategies, such as the anisohydric plasticity of *T. chinensis* versus the isohydric conservatism of *U. pumila*, is crucial for enhancing drought resilience in seasonally arid forests.

## Data Availability

The raw data supporting the conclusions of this article will be made available by the authors, without undue reservation.
